# Four PQQ-Dependent Alcohol Dehydrogenases Responsible for the Oxidative Detoxification of Deoxynivalenol in a Novel Bacterium *Ketogulonicigenium vulgare* D3_3 Originated from the Feces of *Tenebrio molitor* Larvae

**DOI:** 10.3390/toxins15060367

**Published:** 2023-05-30

**Authors:** Yang Wang, Donglei Zhao, Wei Zhang, Songshan Wang, Yu Wu, Songxue Wang, Yongtan Yang, Baoyuan Guo

**Affiliations:** 1 Academy of National Food and Strategic Reserves Administration, Beijing 100037, China; wy@ags.ac.cn (Y.W.);; 2 School of Health Science and Engineering, University of Shanghai for Science and Technology, Shanghai 200093, China

**Keywords:** DON biodegradation, 3-keto-DON, toxicity, *Ketogulonicigenium vulgare*, PQQ-dependent alcohol dehydrogenases

## Abstract

Deoxynivalenol (DON) is frequently detected in cereals and cereal-based products and has a negative impact on human and animal health. In this study, an unprecedented DON-degrading bacterial isolate D3_3 was isolated from a sample of *Tenebrio molitor* larva feces. A 16S rRNA-based phylogenetic analysis and genome-based average nucleotide identity comparison clearly revealed that strain D3_3 belonged to the species *Ketogulonicigenium vulgare*. This isolate D3_3 could efficiently degrade 50 mg/L of DON under a broad range of conditions, such as pHs of 7.0–9.0 and temperatures of 18–30 °C, as well as during aerobic or anaerobic cultivation. 3-keto-DON was identified as the sole and finished DON metabolite using mass spectrometry. In vitro toxicity tests revealed that 3-keto-DON had lower cytotoxicity to human gastric epithelial cells and higher phytotoxicity to *Lemna minor* than its parent mycotoxin DON. Additionally, four genes encoding pyrroloquinoline quinone (PQQ)-dependent alcohol dehydrogenases in the genome of isolate D3_3 were identified as being responsible for the DON oxidation reaction. Overall, as a highly potent DON-degrading microbe, a member of the genus *Ketogulonicigenium* is reported for the first time in this study. The discovery of this DON-degrading isolate D3_3 and its four dehydrogenases will allow microbial strains and enzyme resources to become available for the future development of DON-detoxifying agents for food and animal feed.

## 1. Introduction

Deoxynivalenol (DON) is a natural toxin that is produced as a secondary metabolite by certain species of phytopathogenic fungi belonging to the genus *Fusarium* [1]. Its high prevalence and widespread occurrence in small cereal grains and their derivatives have been extensively documented in the literature [2,3]. The presence of DON in the food supply chain constitutes a notable risk to food safety and public health. A range of adverse health effects, including gastrointestinal disturbances, dermatological manifestations, and immunosuppression, have been associated with varying doses and exposure durations to DON [4,5,6,7]. Given DON’s chemical and thermal stability, salvaging DON-contaminated cereal grains and avoiding the health risks associated with DON is technically challenging. Over recent decades, extensive efforts have been made to seek cost-effective and efficacious DON mitigation strategies. For instance, a range of physicochemical detoxification approaches, such as ultraviolet irradiation [8], argon plasma exposure [9], ozonation treatment [10,11], sorting and separation [12], washing [13,14], and thermal treatment [15] have been explored. However, each of these DON-mitigation methods possesses inherent limitations regarding environmental impacts, nutritional quality, and potential generation of toxic byproducts. The biodegradation method, involving enzymatic transformation of mycotoxins into less toxic or non-toxic products via selected microorganisms, is characterized by mild reaction conditions and a relatively high specificity. Therefore, it was viewed as a promising alternative strategy to reduce DON contamination in agricultural products.

The main toxicity determinants in the molecular structure of DON are the three hydroxyl moieties located at C3, C7, and C15, as well as the epoxy ring connecting C12 and C13 [7,16], with C3-OH and the epoxy moiety as the primary targets for biodegradation. The oxidation of C3-OH can yield 3-keto-DON, which can further undergo epimerization to form 3-epi-DON. Furthermore, de-epoxidation of the epoxy moiety can generate deepoxy-deoxynivalenol (DOM-1). These three metabolites have demonstrated a lower toxicity than DON in vitro [17,18,19], and many species of microorganisms have been found to possess these metabolic capabilities. For instance, several soil-borne bacterial strains, including *Nocardioides* sp. WSN05-2 [20], *Devosia mutans* 17-2-E-8 [21], *Paradevosia shaoguanensis* DDB001 [22], and *Nocardioides* sp. ZHH-013 [23], can produce 3-epi-DON. The two-step enzymatic reaction mechanism converting DON to 3-epi-DON was first elucidated in *D. mutans* 17-2-E-8. The process involves a PQQ-dependent alcohol dehydrogenase (DepA) oxidizing DON to 3-keto-DON, followed by an NADPH dependent aldo-keto reductase (DepB) stereospecifically reducing the intermediate 3-keto-DON to 3-epi-DON [21,24]. Although DON can also be converted to 3-keto-DON by *Agrobacterium* E3-39 [17], *D. insulae* A16 [25], and *Pelagibacterium halotolerans* ANSP101 [26], these strains cannot further convert 3-keto-DON to 3-epi-DON, likely due to the absence of a functional enzyme such as DepB.

Despite numerous successful isolations of DON-degrading microorganisms from various sources, there have been no reports of such isolations from the intestines or feces of insects. As a kind of edible insect, *Tenebrio molitor* larvae (yellow mealworm) are an alternative protein source for food and feed, due to their low production costs and good nutritional characteristics [27]. They can be readily raised on cereal bran or flour; however, these diets may be contaminated with mycotoxins, particularly DON. Long-term feeding of mycotoxin-contaminated diets could thus create favorable conditions for the acclimation of mycotoxin-degrading microorganisms in yellow mealworm. Several investigations have explored the impacts of DON on the biological parameters of insect larvae, as well as the profiles of DON metabolism and excretion [28,29,30]. For example, Van Broekhoven et al. [30] found that DON and its metabolites generally do not remain in the bodies of insect larvae fed diets contaminated with mycotoxins, but can be detected in larval feces, implying that yellow mealworms and/or their gut microbiota may have the potential ability to metabolize DON. On the other hand, microbial resources capable of degrading DON under both anaerobic and aerobic conditions are limited, posing a challenge for the effective mitigation of DON contamination in various application scenarios. In this regard, there is an urgent need to screen for novel DON-degrading strains from diverse sources, to enrich the repository of DON-degrading strains. This can help to address specific application demands in different scenarios, such as agricultural production and animal feed.

In the current study, a novel microbial DON degrader D3_3 was isolated from a sample of yellow mealworm feces using conventional enrichment and isolation procedures. Genome-based ANI analysis and 16S rRNA-based phylogenetic tree analysis consistently revealed its taxonomic position. The impact of various conditions on the strain’s DON-degrading activity was also investigated. Subsequently, the chemical structure of the DON metabolite was determined via mass spectrometry, and its animal cell cytotoxicity and phytotoxicity were evaluated using human gastric epithelial cells (GES-1) and duckweed (*Lemna minor*), respectively. Finally, four PQQ-dependent alcohol dehydrogenases (ADHs) in strain D3_3 were identified as being responsible for the catalysis of the oxidation of DON to 3-keto-DON.

## 2. Results

### 2.1. A Potent DON-Oxidizing Strain Ketogulonicigenium Vulgare D3_3 Isolated from Yellow Mealworm Feces

**Figure 1:**
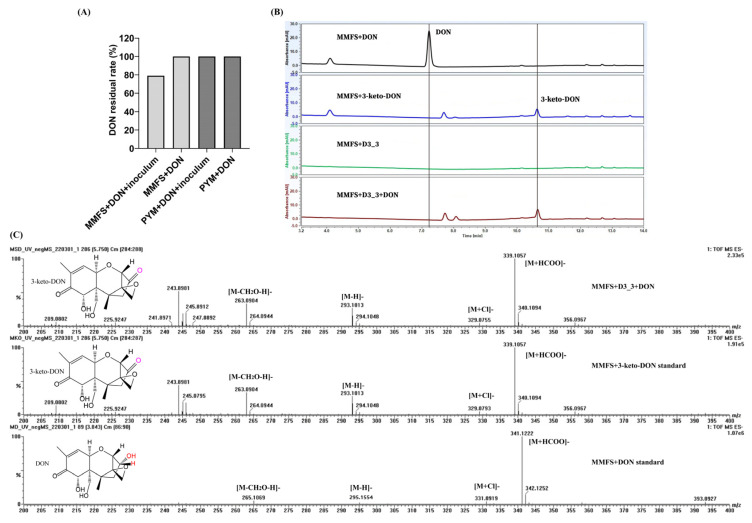
DON degradation profiles of the enrichment cultures and the isolate D3_3’ pure culture, as well as determination of the chemical structure of DON metabolite. (**A**) DON residual rate in MMFS and PYM media containing 50 mg/L of DON with or without insect fecal slurry after four instances of serial subcultivation. (**B**) LC profiles of DON degradation by the isolate D3_3 in MMFS medium containing 50 mg/L of DON. (**C**) MS profiles of DON, 3-keto-DON, and the putative metabolite 3-keto-DON generated by *K. vulgare* strain D3_3.

As demonstrated in Figure 1A, four successive subcultures of yellow mealworm excreta in MMFS (mineral medium supplemented fecal supernatant) liquid resulted in a 20.9% decrease in 50 mg/L DON at 30 °C and 220 rpm in a shaking incubator after 5 d. However, no DON reduction was observed for the sample of PYM that underwent the same enrichment procedure. Thus, the mixed culture with DON-degrading activity was spread on MMFS agar plates after serial dilution. After growing at 30 °C for 7 d, 40 colonies were examined for their ability to remove DON. This resulted in the isolation and selection of the DON-degrading strain D3_3. As demonstrated in Figure 1B, a pure culture of this isolate D3_3 grown in MMFS (initial pH 7) was capable of removing 50 mg/L of DON after 12 h of incubation. Furthermore, a peak in the MMFS+DON+D3_3 sample had the same retention time (Rt = 10.7 min) as the 3-keto-DON standard, which was not detected when D3_3 was incubated in a MMFS medium without DON (MMFS+D3_3), implying that it was a DON metabolite and possibly 3-keto-DON.

The chemical structure of possible 3-keto-DON (Rt = 5.75 min in UPLC-Q-TOF-MS analysis) was further ascertained via MS analysis. Figure 1C reveals that there were several peaks at *m*/*z* 339.1057, 329.0755, 293.1013, and 263.0904 in the negative mode, which were assigned to [M+HCOO]^−^, [M+Cl]^−^, [M-H]^−^, and [M-CH2O-H]^−^, respectively. These data were consistent with those of the 3-keto-DON standard. Furthermore, the MS data of DON (Rt = 3.84 min) showed that a number of peaks, such as *m*/*z* 341.1222 ([M+HCOO]^−^), 331.0919 ([M+Cl]^−^), 295.1154 ([M-H]^−^), and 265.1069 ([M-CH2O-H]^−^), were larger by 2 in comparison to the corresponding ions of the DON metabolites; such an increase was equal to the atomic weight of two hydrogen atoms. These findings conclusively determined that the DON metabolite produced by *K. vulgare* D3_3 was indeed the oxidation product of DON, namely 3-keto-DON.

**Figure 2:**
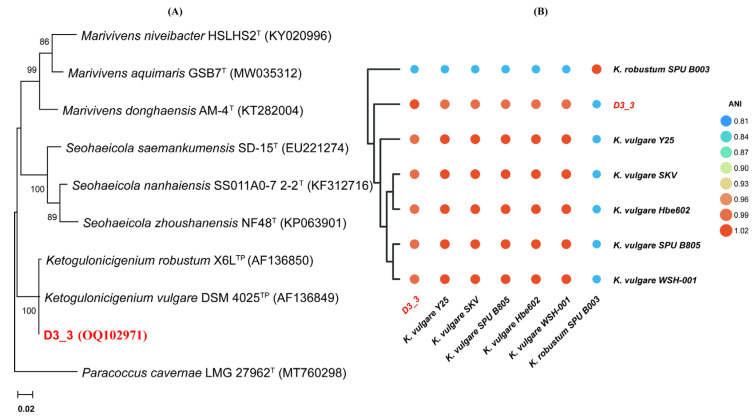
Taxonomic identification of DON-degrading strain D3_3. (**A**) The 16S rRNA-based phylogenetic tree of strain D3_3 reconstructed using the maximum likelihood method. The numbers near the nodes on the phylogenetic tree indicate that bootstrap values greater than 50%, while the content in parentheses are the GenBank accession numbers of the 16S rRNA sequences. Scale bar: 2 nucleotide substitutions per 100 positions. T: type strain, TP: use of patent strain as type strain. (**B**) ANI heatmap of seven *Ketogulonicigenium* strains. Heatmap generated based on ANI matrix obtained from *Ketogulonicigenium* genomes’ average nucleotide identity (ANI) values, ranging from low (blue) to high (orange).

The 16S rRNA gene sequence of strain D3_3 determined in this study comprised 1322 nt (GenBank accession no. OQ102971), exhibiting the highest sequence similarities with that of *Ketogulonicigenium vulgare* DSM 4025^T^ (99.85%) and *Ketogulonicigenium robustum* X6L^T^ (99.47%), respectively. Furthermore, phylogenetic analysis based on a 16S rRNA gene sequence also indicated that strain D3_3 was positioned within the genus *Ketogulonicigenium* and formed a phylogenetic clade with *K*. *vulgare* DSM 4025^T^ and *K*. *robustum* X6L^T^, suggesting that it belonged to the genus *Ketogulonicigenium* (Figure 2A). However, these results could not definitively confirm which species strain D3_3 belonged to. To further clarify its taxonomic status, its genome sequence (obtained from the genome sequencing analysis) and several other genome sequences of *Ketogulonicigenium* available in the NCBI Genome database were subjected to pairwise average nucleotide identity (ANI) calculations using the FastANI algorithm [31]. As shown in Figure 2B, the heatmap indicated that the strain D3_3 shared an ANI of 98.33%, 98.36%, 98.40%, 98.43%, 98.44%, and 81.69% with *K*. *vulgare* Y25 (GCF_000164885), *K*. *vulgare* SKV (GCF_001693655), *K*. *vulgare* SPU B805 (GCF_001855295), *K*. *vulgare* Hbe602 (GCF_001399515), *K*. *vulgare* WSH-001 (GCF_000223375), and *K*. *robustum* SPU B003 (GCF_002117445), respectively. According to the classification criteria of >95% intra-species and <83% inter-species ANI values [31], the DON-degrading strain D3_3 was definitively identified as *K*. *vulgare.*

### 2.2. Effects of Different Growth Factors on the DON-Degrading Activity of Strain D3_3

**Figure 3:**
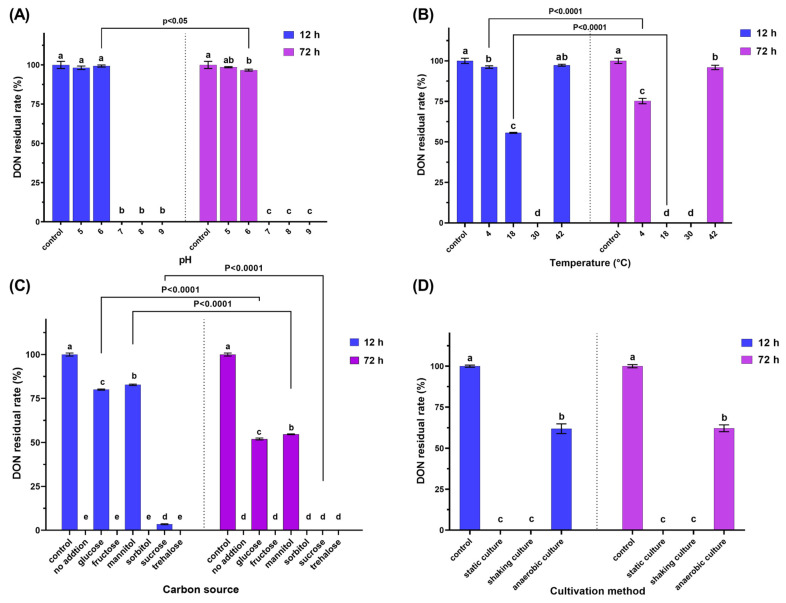
The impacts of different factors on the DON-degrading activity of strain D3_3 in the MMFS medium containing 50 mg/L of DON at incubation times of 12 and 72 h. (**A**) pH, (**B**) temperature, (**C**) carbon source, and (**D**) cultivation method. Significant differences (*p* < 0.05) in the degradation rates between the two groups, as determined via ordinary one-way ANOVA with a use of Tukey’s multiple comparisons test, are indicated by the different letters (a–e) above the columns. “Control” stands for the MMFS medium containing 50 mg/L of DON, while “no addition” stands for the DON-added and inoculated MMFS medium.

As shown in Figure 3A, residual DON was not detected after 12 h in samples with an initial pH of 7, 8, and 9, meanwhile the DON residual rate was not significantly different for the pH 5 and 6 groups compared to the control. After 72 h, the DON residual rate of pH 6 decreased to 96.7 ± 0.6%, exhibiting a significant contrast with the control and the 12 h rate for the same group (*p* < 0.05). However, no such change was observed for the pH 5 group. Conclusively, strain D3_3 can degrade DON with a pH range of between 6 and 9, with greater activity between pH 7 and 9 than at pH 6, but no activity at pH 5.

As illustrated in Figure 3B, the 12 h DON residual rates for the samples at 4, 18, 30, 37, and 42 °C were 96.2 ± 0.8, 55.6 ± 0.3, 0, 97.6 ± 0.9, and 98.0 ± 0.6%, respectively. After 72 h, the DON residual rates at 4 and 18 °C decreased significantly to 75.2 ± 1.7 and 0% (*p* < 0.0001), respectively, while the rates at 37 and 42 °C remained statistically similar to their 12 h counterparts. Overall, strain D3_3 exhibited DON-degradation activity at temperatures ranging from 4 to 30 °C, with an optimal temperature of 30 °C. No DON-degradation activity was observed at 37 °C or higher, which is logically understandable considering that the strain does not grow at temperatures above 37 °C. Notably, even at 4 °C, D3_3 still displayed a 24.8 ± 1.7% DON-degradation rate after 72 h.

As shown in Figure 3C, the addition of glucose, mannitol, and sucrose significantly enhanced the DON residual rate at 12 h (*p* < 0.05), with glucose having the greatest effect, followed by mannitol and sucrose. Fructose, sorbitol, and trehalose had no significant effect on the DON residual rate. After 72 h, the DON residual rate for samples with glucose, mannitol, and sucrose declined significantly compared to the respective 12 h treatments (*p* < 0.001). Overall, the D3_3 strain’s ability to degrade DON was inhibited by glucose, mannitol, and sucrose, but not by fructose, sorbitol, and trehalose.

As demonstrated in Figure 3D, the 12 h DON residual rates for samples under static, shaking, and anaerobic conditions were 0, 0, and 61.9 ± 2.9%, respectively. Despite extending the incubation duration to 72 h, the anaerobic DON residual rate remained virtually unchanged (62.1 ± 2.0%). These findings indicate that strain D3_3 possesses the capacity to degrade DON under both anaerobic and aerobic conditions, with the latter being more favorable for DON degradation. This expands this strain’s potential application scenarios, such as being adapted to be used in agricultural practice in aerobic condition, in addition to being used as a feed additive administered to the anaerobic gastrointestinal tract of animals.

### 2.3. Cytotoxicity and Phytotoxicity of 3-Keto-DON

#### 2.3.1. Effect of 3-Keto-DON on the Viability of GES-1 Cells

**Figure 4:**
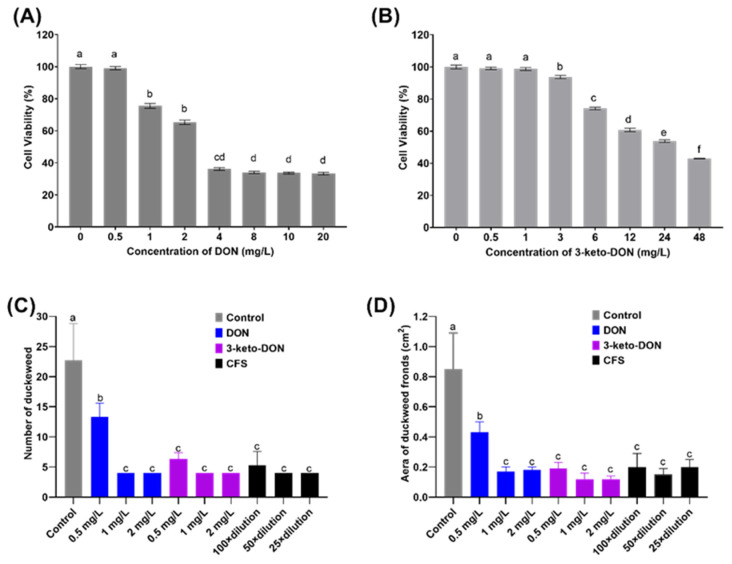
Toxicity evaluation of DON and 3-keto-DON. Effect of different concentrations of DON (**A**) and 3-keto-DON (**B**) on the viabilities of GES-1 cells. Phytotoxicity assessment of DON, 3-keto-DON, and cell-free supernatant of DON-degradation culture (CFS) on the number (**C**) and area (**D**) of duckweed fronds. The use of different lowercase letters (a–f) indicates that there are significant differences (*p* < 0.05) between the treatments being compared. Values that share the same letter are not significantly different.

To gain a better understanding of the toxicity of 3-keto-DON, GES-1 was used as an in vitro model to investigate its toxicity. The results, as illustrated in Figure 4A,B, showed that both DON and 3-keto-DON reduced the viability of GES-1 cells in a dose-dependent manner, with GES-1 being more sensitive to DON than 3-keto-DON at the same treatment dose. The calculated IC_50_ values for DON and 3-keto-DON against GES-1 cells were 2.66 and 29.70 mg/L, respectively, indicating an 11.1-fold decrease in the toxicity of 3-keto-DON against GES-1 cells compared to the parent mycotoxin DON.

#### 2.3.2. Effect of 3-Keto-DON on L. Minor

As shown in Figure 4C, 0.5 mg/L of DON standard significantly decreased the number of fronds by 41.4% when compared to the control (13.3 ± 2.3 vs. 22.7 ± 6.1 fronds/well), while the area of fronds was lowered by 49.4% (0.43 ± 0.07 vs. 0.85 ± 0.24 cm^2^/well). Duckweed development was entirely repressed by DON at 1 mg/L or above, with no increase in both the area and number of fronds after 7 d of exposure.

As demonstrated in Figure 4D, 0.5 mg/L of the 3-keto-DON standard resulted in a considerable decrease of 72.2% in the number of fronds (6.3 ± 1.1 compared to 22.7 ± 6.1 fronds/well in the control) and a 77.7% decrease in area (0.19 ± 0.04 compared to 0.85 ± 0.24 cm^2^/well in the control), both of which were statistically significant (*p* < 0.05). When compared to 0.5 mg/L of DON standard, the number and area of fronds were reduced by 52.6% (6.3 ± 1.1 vs. 13.3 ± 2.3 fronds/well) and 55.8% (0.19 ± 0.04 vs. 0.43 ± 0.07 cm^2^/well), respectively, with statistical significance (*p* < 0.05). Furthermore, it was observed that only a small number of duckweed fronds were bleached when exposed to 3-keto-DON at 0.5 mg/L. In comparison, all of the fronds became bleached when exposed to 1 and 2 mg/L of the compound. However, the phenomenon of fronds becoming bleached did not occur at DON concentrations of 0.5 mg/L or higher.

Prior to the phytotoxicity experiment, UPLC analysis of MMSF+D3_3+DON revealed that DON had been completely converted to 3-keto-DON at a concentration of around 50 mg/L. Moreover, 100-fold, 50-fold, and 25-fold dilutions of the sample yielded 0.5, 1, and 2 mg/L of 3-keto-DON, respectively. Exposure of duckweed to the 100-fold dilution resulted in a 76.7% reduction in number of fronds (5.3 ± 2.3 vs. 22.7 ± 6.1 fronds/well in the control) and a 76.5% reduction in the area of fronds (0.2 ± 0.09 vs. 0.85 ± 0.24 cm^2^/well in the control), while 50-fold and 25-fold dilutions completely inhibited growth. No significant differences in growth inhibition were observed between the 100-fold dilution of MMSF+D3_3+DON and 0.5 mg/L of the 3-keto-DON standard, or between the 50-fold and 25-fold dilutions and 1 and 2 mg/L of the 3-keto-DON standard, respectively (*p* > 0.05). According to these results, we can conclude that the phytotoxicity of 3-keto-DON seems to be greater than that of the same amount of DON.

### 2.4. Four PQQ-Dependent Alcohol Dehydrogenases Responsible for DON Transformation

To gain a greater understanding of *K. vulgare* strain D3_3 and screen its DON-oxidizing genes, its genome was sequenced, assembled, and then annotated. The analysis of D3_3’s genome revealed that it comprised one circular chromosome and three plasmids, with a total length of 3,293,003 bp and a GC content of 61.36%. PGAP annotation predicted 3236 putative protein-coding genes, 5 rRNA operons, and 60 tRNAs (Table S1).

Currently, there are two different types of sequence-known enzymes that can oxidize the hydroxyl at C3 of DON into a keto group, namely an aldo-keto reductase AKR18A1 from *Sphingomonas* sp. S3-4 and two pyrroloquinoline quinone (PQQ)-dependent dehydrogenases DepA and QDAH from *D. mutans* 17-2-E-8 and *Devosia* sp. D6_9 [24,32,33]. Since DepA and QDAH share the same amino acid sequences, only DepA and AKR18A1 were used as query protein sequences to search for potential genes involved in DON oxidation against the resulting genome database. Based on BLASTp search outcomes, the genome contained fourteen potential genes for DON oxidation, comprising eight ADH-encoding genes and six AKR-encoding genes. The protein sequences encoded by these genes displayed amino acid sequence similarities to DepA and AKR18A1, ranging 24.8–57.8% and 28.2–34.7%, respectively (Tables S2 and S3). Furthermore, six of the eight dehydrogenase-encoding genes were located on a 2.8 Mb bacterial chromosome, whereas the other two were separately found on two 0.22 Mb megaplasmids of pP1 and pP2 (Table S2). Five of the six aldo-keto reductase-encoding genes were located on the bacterial chromosome, and the other one was found on megaplasmid pP1 (Table S3).

**Figure 5:**
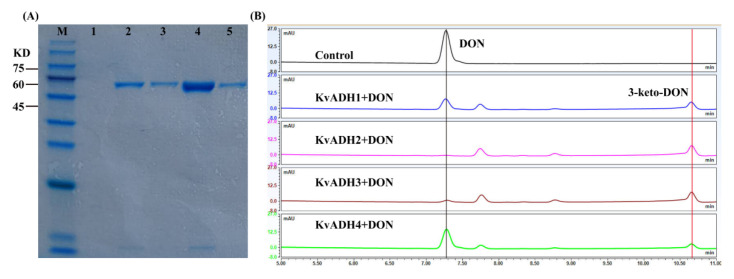
Molecular weight characterization and enzymatic activity confirmation of four recombinant PQQ-dependent alcohol dehydrogenases. (**A**) SDS-PAGE analysis of four Ni-affinity purified recombinant ADHs. M: molecular weight markers (10–180 KD); Lane 1: control; Lane 2: *Kv*ADH1; Lane 3: *Kv*ADH2; Lane 4: *Kv*ADH3; Lane 5: *Kv*ADH4. (**B**) LC profiles of 3-keto-DON produced via in vitro DON oxidation using four ADHs with PQQ, Ca^2+^, and PMS present.

The signal peptide sequences were predicted and then deleted prior to heterogeneously expressing candidate enzymes. As predicted using SignalP 5.0, seven of the eight dehydrogenases possessed a signal sequence ranging from 21 to 24 amino acids in length, whereas all aldo/keto reductases did not possess it (Tables S2 and S3). Cloning, expression, and confirmation of enzymatic activity were performed on the fourteen candidate genes. As shown in Figure 5A, four of the eight ADHs, designated *Kv*ADH1, *Kv*ADH2, *Kv*ADH3, and *Kv*ADH4, yielded a single band at a molecular mass of 61.7, 61.2, 61.3, and 61.6 kDa, respectively. *Kv*ADH1, *Kv*ADH2, *Kv*ADH3, and *Kv*ADH4 showed 57.76%, 55.14%, 54.87%, and 55.86% amino acid similarity with DepA or QDDH, respectively; the amino acid sequence identities between the four ADHs obtained in this study ranged from 80.7 to 86.5% (Table S4). The four purified *Kv*ADHs exhibited catalytic activity toward DON in the presence of the cofactors PQQ and CaCl_2_, as well as the artificial electron acceptor PMS (Figure 5B), but no activity was observed without PQQ being present. It is therefore suggested that PQQ is necessary for maintaining the enzymatic function of the four dehydrogenases. Additionally, none of the six AKRs had such enzymatic activity in the presence of NADH or NADPH. These results clearly demonstrated that the oxidation of DON to 3-keto-DON in the *K. vulgare* strain D3_3 was caused by the PQQ-dependent alcohol dehydrogenase rather than the aldo-keto reductase.

## 3. Discussion

Many species of DON-degrading microbes have so far been successfully isolated from a variety of sources, such as wheat leaf, wheat head, soil, lake water, seawater, various animal intestines, bovine rumen, and human milk [20,22,34,35,36,37,38,39,40,41,42,43,44]. Nevertheless, there have been no reports regarding the isolation of a DON-degrading strain from yellow mealworm. Herein, we successfully isolated a DON-degrading strain *K. vulgare* D3_3 from a single insect feces sample. Additionally, three DON-degrading *Devosia* strains were effectively screened from five other yellow mealworm feces samples collected from different geographical areas [45]. These results exemplify that yellow mealworm feces are a highly efficient source for the isolation of mycotoxin-degrading microorganisms.

Furthermore, this is the first report on a member of the genus *Ketogulonicigenium* as a potent DON-degrading microorganism. *K. vulgare*, the type species of the genus *Ketogulonicigenium*, was first isolated from soil and taxonomically characterized in 2001 [46]. It is currently used in the microbial production of a key intermediate for the industrial synthesis of vitamin C, namely 2-keto-L-gulonic acid [47]. Due to its unique nature and lack of many amino acid biosynthesis pathways, *K. vulgare* grew poorly, even on nutrient-rich media [48]. The isolate D3_3 also exhibited this poor growth phenotype, with a maximum optical density (OD_600_) of 0.13 (4 × 10^6^ CFU/mL) in MMFS medium and only 0.25 in nutrient-rich TSB medium after 72 h incubation. However, it exhibited a highly efficient DON-degradation rate of 104.1 μg/h/per 10^7^ cells, exceeding the degradation rates reported in earlier studies for *D. mutans* 17-2-E-8, *P. shaoguanens* is DDB001, and *Devosia* sp. D6-9, which had degradation rates of 0.3, 6.8, and 15.0 μg/h/per 10^7^ cells, respectively [22,33,37]. In addition, the versatile DON-degrading capabilities of the facultative anaerobic strain D3_3 under aerobic or anaerobic conditions greatly expand its potential application scenarios.

3-keto-DON is a common intermediate or end metabolite in microbial DON degradation. Several strains of bacteria, including *Agrobacterium-Rhizobium* E3-39, *D. insulae* strain A16, and *P. halotolerans* ANSP101 can directly convert DON to 3-keto-DON [17,25,26]. Other strains, including *D. mutans* 17-2-E-8, *Devosia* sp. D6-9, *Lactobacillus rhamnosus* SHA113, *Nocardioides* sp. WSN05-2, *Nocardioides* sp. ZHH-013, and *Sphingomonas* sp. S3-4, could first convert DON to 3-keto-DON, which was then stereospecifically reduced to 3-epi-DON [23,32,33,37,42,43]. The absence of 3-epi-DON in the MMFS+DON+D3_3 sample indicated that *K. vulgare* D3_3 was incapable of reducing 3-keto-DON to 3-epi-DON, likely due to a lack of the responsible enzyme.

The bacterium *K. vulgare* D3_3 could transform DON to 3-keto-DON. However, before considering its potential use, it is essential to evaluate the toxicity of its transformation product. Currently, there is a scarcity of toxicity data for 3-keto-DON, with only two studies assessing its in vitro cytotoxicity on human colon cancer cells (Caco-2) and mouse spleen lymphocytes [17,49], and data on cytotoxicity against GES-1 cells are also lacking. In this study, the calculated IC_50_ values for DON and 3-keto-DON against GES-1 cells were 2.66 and 29.70 mg/L, respectively. However, in a previously described MTT (3-[4,5-dimethylthiazol-2-yl]-2,5 diphenyl tetrazolium bromide) bioassay assessing Caco-2 cell viability, the IC_50_ values for 3-keto-DON and DON were 1.24 mg/L and 0.409 mg/L, respectively [49]. It is considered that the discrepancy in IC_50_ values between the two studies mainly resulted from different cell lines (GES-1 vs. Caco-2) and assay methodologies (CKK-8 vs. MTT). In addition, taking into account the fact that the IC_50_ value (2.66 mg/L) of DON in this study is analogous to the IC_50_ value (2.99 mg/L) published in earlier studies [50] using the same cell lines and detection methods, the validity of the IC_50_ values of DON and 3-keto-DON in the current study should be verified. In summary, our study provides initial evidence that 3-keto-DON is less toxic to GES-1 compared to its parent mycotoxin DON, similarly to other cell lines.

PQQ-dependent alcohol dehydrogenases represent a class of oxidoreductases that utilize the cofactor PQQ to catalyze the oxidation of a variety of substrates, including alcohols and sugars [51,52,53]. Based on their tertiary structures, PQQ-dependent ADHs can be categorized into two families: six-bladed “propeller fold” and eight-bladed “propeller fold” [54]. The latter can be further classified into three types based on their location and structural characteristics: type I (periplasmic, dimeric), type II (periplasmic, monomeric, with a heme c group), and type III (membrane-bound, dimeric or trimeric, with multiple heme c groups) [55]. The crystal structures of many different types of PQQ-dependent ADHs have been determined, providing structural foundations for understanding the substrate–cofactor–enzyme binding and electron transfer mechanisms. Recently, the crystal structures of the first identified PQQ-dependent DON dehydrogenase, DepA (PDB:7WMD), and its complex with PQQ (PDB:7WMK), were determined, suggesting that DepA belongs to the Type I PQQ-dependent ADH [56]. These crystal structures, along with biochemical evidence, confirm the interactions between DepA, PQQ, and DON, and reveal a unique tyrosine residue crucial for substrate selection. In this study, four PQQ-dependent alcohol dehydrogenases (*Kv*ADHs) with DON oxidation activity in the genome of strain D3_3 were identified. These four enzymes exhibit high amino acid homology (54.87–57.76%) with DepA and 80.7–86.5% homology with each other. This suggests that the four *Kv*ADHs and DepA may share a similar catalytic mechanism, but with some differences in their amino acid sequences that could indicate differences in substrate specificity or other functional properties. Further research is required to elucidate the structure and function of the four *Kv*ADHs, including studies which involve the crystallization and determination of their three-dimensional structures, as well as observations of their kinetic properties with DON and other alcohol substrates. In addition, some bacteria, such as *Pseudomonas*, methanotrophic, and methylotrophic bacteria, generally express different categories and even multiple PQQ-ADHs of the same type, demonstrating the importance of these enzymes for the metabolism of various alcohol substrates [52,57,58]. Within D3_3, there are eight putative PQQ-dependent dehydrogenases whose functional redundancy may enhance the adaptability of microorganisms in maintaining critical functions under fluctuating environmental conditions and diverse microbial communities.

Despite challenges such as low bacterial cell yields, high-cost cofactor PQQ requirements, and the weak robustness of wild enzymes, optimizing the cultivation conditions to improve bacterial cell yield and engineering the enzyme to enhance its robustness could help overcome these limitations and maximize the potential of the isolate and enzyme for industrial applications.

## 4. Conclusions

In this study, a novel DON-degrading bacterial strain, *K. vulgare* D3_3, was isolated from yellow mealworm feces and was found to completely degrade 50 mg/L of DON within 12 h under optimal conditions of pH 7.0–9.0, 30 °C, and anaerobic cultivation. The metabolite 3-keto-DON displayed lower cytotoxicity against GES-1 cells than its parent mycotoxin DON, but greater phytotoxicity toward duckweed. Additionally, four genes encoding PQQ-dependent dehydrogenases in the genome of isolate D3_3 were identified as being responsible for catalyzing the oxidation of DON. These findings suggest that the strain and enzyme have potential to be developed as detoxification agents to address DON contamination in food and animal feed, despite certain challenges, such as the low yields of bacterial cells, high-cost cofactor PQQ, and weak robustness of the wild-type enzyme.

## 5. Materials and Methods

### 5.1. Chemicals and Reagents

Standard DON and 3-keto-DON, both with a purity of 98%, were bought from Pribolab Pte. Ltd. (Qingdao, China) and TripleBond Corporation (Guelph, ON, Canada), respectively. The methanol and acetonitrile for ultra-performance liquid chromatography (UPLC) and UPLC coupled to quadrupole time-of-flight mass spectrometry (UPLC-Q-TOF-MS) analysis were of chromatographic grade and were purchased from Fisher Scientific International Inc. (Pittsburgh, PA, USA). All other chemicals and reagents were analytical-grade and obtained from various commercial sources.

### 5.2. Enrichment, Isolation, and Identification of DON-Degrading Microorganisms

#### 5.2.1. Yellow Mealworm Feces Collection and Processing

Approximately 1 kg of yellow mealworms were bought from a pet supply market located in the Daxing District of Beijing. Upon arrival at the laboratory, they were placed in a pre-sterilized plastic container and fasted for 48 h, to empty their gastrointestinal tract of feed and produce an abundance of fecal pellets. These pellets were sifted from the yellow mealworms using a 40-mesh sieve and harvested with a sterile sampling bag. Then, 25 g of fresh feces was weighed and immersed in 500 mL of aseptic phosphate buffer (50 mM, pH 7) in a 2 L sterile Erlenmeyer flask. The mixture was homogenized via shaking for 30 min at 220 rpm and 30 °C, to make a 5% (*w*/*v*) fecal slurry. A small portion of this slurry served as the inoculum for subsequent enrichment culture experiments. The remaining slurry was centrifuged and subsequently filter sterilized. The resulting supernatant, namely the cell-free yellow mealworm fecal supernatant (FS), was stored at −20 °C for further use.

#### 5.2.2. Enrichment and Isolation Procedures

For the enrichment and isolation of DON-degrading microorganisms, two different media, namely MMFS and PYM, were employed. The MMFS medium comprised Na_2_HPO_4_·12H_2_O (4.03 g/L), KH_2_PO_4_ (1 g/L), KNO_3_ (0.59 g/L), (NH_4_)_2_SO_4_ (0.5 g/L), MgSO_4_·7H_2_O (0.5 g/L), CaCl_2_ (0.02 g/L), FS (10:90, *v*/*v*), and 1× trace metal mixture [59]; while the PYM medium contained peptone (10 g/L), yeast extract (2 g/L), and MgSO_4_·7H_2_O (1 g/L). For the enrichment procedure, 5 mL of each medium pre-spiked with 50ppm DON was inoculated with and without 500 μL fecal slurry as the treatment samples and control samples, respectively. These cultures were continuously shaken at 220 rpm under 30 °C for 5 d. After the initial incubation, two cultures were subcultured four times in the respective fresh media at an inoculum ratio of 1:100. Residual DON in the final enrichment cultures was assessed via UPLC, to ascertain DON-degrading capabilities. The positive culture was diluted and plated, and randomly picked single colonies were then cultivated individually in DON-containing liquid media for 3 d before being analyzed for residual DON via UPLC to obtain the DON-degrading microbial strain.

#### 5.2.3. 16S rRNA-Based Phylogenetic Analysis of DON-Degrading Strain

A 16S rRNA-based phylogenetic analysis was conducted to determine the taxonomic position of the DON-degrading strain. Briefly, a partial 16S rRNA gene fragment was amplified via PCR using universal primers 27F (AGAGTTTGATYMTGGCTCAG) and 1492R (CGGYTACCTTGTTACGACTT). Then, the amplified product was sequenced and compared with sequences in the 16S ribosomal RNA database to obtain evolutionarily closely related 16S rRNA gene sequences using the BLASTn program. Finally, a phylogenetic tree was built using the maximum likelihood method with MEGA 11.0 software [60].

### 5.3. Effects of Various Factors on the DON-Degrading Activity of Strain D3_3

In a degradation system consisting of 2 mL of MMFS medium, 50 mg/L DON, and 8 × 10^5^ CFU/mL of initial cell concentration (OD = 0.025), we investigated the effects on degradation activity as a result of four factors: incubation temperature (4, 18, 30, 37, and 42 °C); initial pH (5.0, 6.0, 7.0, 8.0, and 9.0); addition of 2% (*w*/*v*) sugar and sugar alcohol (glucose, fructose, mannitiol, sorbitol, sucrose, and trehalose); and cultivation conditions (shaking at 220 rpm, static, and anaerobic). During the investigation of the impact of a single factor, the other parameters were maintained constant at 30 °C, pH = 7.0, no sugar alcohol addition, and shaking at 220 rpm. A control group was established using MMFS with DON but without inoculum. Each treatment was conducted in triplicate and its residual DON was analyzed using the UPLC technique at 12 and 72 h. The DON residual rate (%) was calculated by measuring the peak area of DON (UV absorption at 220 nm) and using the following equation:

(1) DON residual rate=Asample/Acontorl×100%

where A_sample_ and A_contorl_ are the values of the sample and control, respectively.

### 5.4. DON and Its Metabolite Analysis

#### 5.4.1. DON Detection Using UPLC Technique

A Thermo Scientific Dionex UltiMate 3000 system comprising a quaternary RS pump, a column oven, an autosampler, a diode array detector, and a system controller software Chromeleon 7.2 was used for UPLC analysis. First, 2 μL of sample was injected into an Acquity BEH C18 column (1.7 μm, 100 mm × 2.1 mm) and analyzed under the conditions of 40 °C, 0.2 mL/min flow rate, and a 220 nm detection wavelength. The elution gradients of solvent A (water) and solvent B (acetonitrile) were used as follows: 0–6 min, gradient 5% to 25% B; 6–12 min, isocratic 25% B; 12–13 min gradient 25% to 5% B; 13–18 min, isocratic 5% B. 

#### 5.4.2. Analysis of DON Degradation Metabolite Using the UPLC-Q-TOF-MS Method

The DON metabolite was analyzed using a Waters Xevo G2-S quadrupole time-of-flight mass spectrometer with an electrospray ionization source (negative ion mode), coupled to a Waters Acquity UPLC system. Liquid chromatography was performed similarly to the DON detection method 5.4.1, with the exception of using a different gradient elution program with solvent A (water containing 5 mM ammonium formate) and solvent B (methanol) as follows: 0–1 min, isocratic 10% B; 1–19 min, gradient 10–90% B; 19–24 min, isocratic 90% B; 24–25 min, gradient 90–10% B; 25-30 min, isocratic 10% B. The ionization source conditions were set as follows: 2 kV capillary voltage; 450 °C desolvation temperature; 800 L/h desolvation gas (N_2_); 120 °C source temperature; and 50 L/h cone gas (N_2_).

### 5.5. Toxicity Assay of DON and Its Metabolite

#### 5.5.1. In Vitro Cytotoxicity Assay Using GES-1

The in vitro toxicity of DON and its oxidation product (3-keto-DON) were investigated using GES-1 cells and a CKK-8 test. GES-1 cells were bought from Cobioer Biosciences Co., Ltd. (Nanjing, China), and maintained under the cultivation conditions reported by Yang et al. [50]. First, 10,000 cells/well were plated into 96-well plates and allowed to proliferate to 80% confluence before cells were collected and exposed to 3-keto-DON (0.5, 1, 3, 6, 12, 24, and 48 mg/L) and DON standard (0.5, 1, 2, 4, 8, 10, and 20 mg/L) for 24 h. Following that, the spent medium was exchanged with a fresh one containing 1 mg/mL of CCK-8, which was then incubated for 4 h. Finally, cell viability was determined using an ELISA reader to measure the optical density at 450 nm. The percentage inhibition compared to control-treated cells was calculated for each compound concentration. All analyses were performed in triplicate. Statistical differences were determined using one-way ANOVA, with *p* < 0.05 considered to be statistically significant. IC_50_ values were calculated using Dr Fit software version 1.042 [61] with default setting parameters.

#### 5.5.2. Phytotoxicity Assay Using *Lemna minor*

To evaluate the phytotoxicity of 3-keto-DON, the growth of *Lemna minor* (common duckweed) was examined after exposure to test samples. Duckweed was collected from a pond in Daxing District, Beijing, China, disinfected, and acclimated to experimental conditions according to the method described by Megateli et al. [62]. Four duckweed fronds were aseptically inoculated into each well of 24-well plates preloaded with 2 mL of media supplemented with different test samples, including working concentrations of 100-fold, 50-fold, and 25-fold dilutions of MMFS+D3_3+DON (derived from cell-free culture supernatant of strain D3_3 grown for 3 d in MMFS medium supplemented with 50 mg/L of DON); 0.5, 1, and 2 mg/L of DON and 3-keto-DON standard solution; and no supplement. All treatments were performed in triplicate. After 7 d of incubation, the number of fronds was counted with a microscope. Each well was photographed, and the frond area was calculated using ImageJ software version 1.53p.

### 5.6. Identification of DON-Oxidizing Enzyme in Strain D3_3

#### 5.6.1. Sequencing, Assembly and Annotation of D3_3’ Genome, as well as Scouting Potential Genes Involved in DON Oxidation

Genome sequencing was carried out at Biomaker Technology Inc. (Beijing, China) using a combination of the Nanopore PromethION 48 system and the Illumina NovaSeq 6000 platform, and technical details on sequencing, assembly, and annotation can be found in our previous report [63].

To screen the candidate enzymes responsible for DON degradation, the two protein sequences of DepA (GenBank accession no. KFL25551.1) and AKR18A1 (GenBank accession no. ASY03293.1), which have been reported to have the catalytic function of oxidizing DON into 3-keto-DON, were individually used as queries against the genome sequence of D3_3 for homology search using BLASTp with an E-value of 10^−6^ using TBtools version 1.098769 [64]. Based on the results of the homology search, superfamily classifications for these candidate proteins were predicted using Superfamily 2.0 [65,66], and their signal peptides were predicted with SignalP-5.0 [67], prior to recombinant protein expression. Multiple sequence alignment of all protein sequences was performed using Clustal Omega software version 1.2.2 [68].

#### 5.6.2. Cloning, Expression, and Activity Assay for Potential DON-Oxidizing Enzymes

To verify whether the candidate enzymes suggested by the homology search results really had the function of degrading DON, we purified fourteen candidate enzymes heterologously overexpressed in *E. coli* and tested their DNA degradation function. For the DNA cloning experiment, the fourteen candidate genes encoding mature enzymes and linearized expression vector pET28a were amplified via PCR using Q5 High-Fidelity DNA Polymerase (NEB), and the detailed information of the PCR primers and cycling conditions are listed in Table S5. The fourteen DNA inserts were then individually cloned into linearized vector pET28a via the T5 exonuclease-dependent DNA assembly (TEDA) method [69], and the resulting ligation reaction mixtures were individually transformed into the expression host *E.coli* BL21(DE3)pLysS for expression as N-terminal 6-His-tag fusion proteins. Recombinant proteins were expressed in an autoinduction medium at 18 °C, according to the previously reported method [59], and purified using PureCube Ni-NTA Agarose (Cube Biotech), according to the manufacturer’s instructions. Purified recombinant protein was assessed using SDS-PAGE and Coomassie Brilliant Blue R-250 staining. Regarding the enzymatic assay for alcohol dehydrogenase, 20 µL of each of the purified recombinant proteins was added to 180 µL of a reaction system containing 1mM Ca^2+^, 100 µM PQQ·Na_2_, 400 µM phenazine methosulfate (PMS), and 50 mg/L DON in 50 mM Tris-HCl buffer (pH 8). Aldo-keto reductase (AKR) activity was determined according to the method reported by He et al. [32]. After 1 h, enzyme-catalyzed reactions were stopped by adding 200 µL of methanol to the reaction systems followed by UPLC analysis.

## Data Availability

The full genomic sequence of *K. vulgare* D3_3 has been deposited in NCBI/GenBank under BioProject number (PRJNA875257) with the GenBank accession numbers (CP103997, CP103998, CP103999, and CP104000) and BioSample number (SAMN30609498). The 16S rRNA gene was deposited under the GenBank accession number OQ102971. Additionally, the data supporting the findings of this work are available within the paper and its Supplementary Information.

## Supplementary Materials

The following supporting information can be downloaded at: https://www.mdpi.com/article/10.3390/toxins15060367/s1, Table S1: Genomic characteristics of *Ketogulonicigenium vulgare* D3_3; Table S2: Eight candidate genes for oxidation of C3-OH group of DON; Table S3: Six candidate genes for oxidation of C3-OH group of DON; Table S4: Percent identity matrix of amino acid sequences created with the Clustal Omega program; Table S5: DNA primers and PCR conditions.
