# Supplementary material for: Four PQQ-Dependent Alcohol Dehydrogenases Responsible for the Oxidative Detoxification of Deoxynivalenol in a Novel Bacterium Ketogulonicigenium vulgare D3_3 Originated from the Feces of Tenebrio molitor Larvae
Source: Toxins (Basel). 2023 May 30;15(6):367. doi: 10.3390/toxins15060367 (PMC10301637; doi:10.3390/toxins15060367)
Supplement: Supplementary file 1 [file toxins-15-00367-s001.zip › toxins-2413584-supplementary-done.pdf]

toxins

MDPI

Supplementary Materials: Four PQQ-Dependent Alcohol Dehydrogenases Responsible for the Oxidative Detoxification of Deoxynivalenol in a Novel Bacterium *Ketogulonicigenium vulgare* D3_3 Originated from the Feces of *Tenebrio molitor* Larvae

Yang Wang, Donglei Zhao, Wei Zhang, Songshan Wang, Yu Wu, Songxue Wang, Yongtan Yang and Baoyuan Guo

Table S1. Genomic characteristics of *Ketogulonicigenium vulgare* D3_3.

Genome Organization	Size [bp]	Genome Size [bp]	G+C content [%]	protein-coding genes	rRNA operons	tRNA genes
Chromosome	2,815,180	3,293,003	61.36	3,236	5	60
Megaplasmid pP1	225,629					
Megaplasmid pP2	216,683					
Plasmid pP3	35,511					

Table S2. Eight candidate genes for oxidation of C3-OH group of DON.

Name of Subject Gene	Score (bits)	E Value	Identities with Mature DepA (%)	Coverage (%)	Location	ORF Position in Genome (bp)	Proenzyme (aas)	Signal Peptide	Superfamily
Kvadh1	655.2	0.00E+00	57.8	95.8	chromosome	964,595–966,334	579	1–23	Quinoprotein alcohol dehydrogenase-like
Kvadh2	638.6	0.00E+00	55.1	96.2	chromosome	1,783,304–1,785,043	579	1–23	Quinoprotein alcohol dehydrogenase-like
Kvadh3	625.2	0.00E+00	54.9	95.7	chromosome	220,372–222,108	578	1–23	Quinoprotein alcohol dehydrogenase-like
Kvadh4	611.7	0.00E+00	55.9	95.8	chromosome	987,889–989,628	579	1–23	Quinoprotein alcohol dehydrogenase-like
Kvadh5	339.3	6E-103	35.3	96.2	chromosome	2,597,007–2,599,091	694	1–24	Quinoprotein alcohol dehydrogenase-like
Kvadh6	156.0	1.00E-11	24.8	56.5	chromosome	192,917–195,370	817	No	Quinoprotein alcohol dehydrogenase-like
Kvadh7	296.2	4.00E-88	34.8	96.7	megaplasmid pP2	128,094–129,782	562	1–22	Quinoprotein alcohol dehydrogenase-like
Kvadh8	211.8	2.00E-59	47.6	36.6	megaplasmid pP1	164,715–165,458	247	1–21	Quinoprotein alcohol dehydrogenase-like

Table S3. Six candidate genes for oxidation of C3-OH group of DON.

Name of Subject Gene	Score (bits)	E Value	Identities with Mature DepA (%)	Coverage (%)	Location	ORF Position in Genome (bp)	Proenzyme (aas)	Signal Peptide	Superfamily
Kvakr1	153.7	4.00E-42	34.0	94.5	chromosome	219,153–220,136	327	No	NAD(P)-linked oxidoreductase
Kvakr2	129.8	7.00E-34	34.7	86.0	chromosome	328,678–329,637	319	No	NAD(P)-linked oxidoreductase
Kvakr3	121.3	5.00E-31	33.6	93.0	chromosome	2,698,903–2,699,943	346	No	NAD(P)-linked oxidoreductase
Kvakr4	120.2	1.00E-30	30.5	90.1	chromosome	230,248–231,213	321	No	NAD(P)-linked oxidoreductase
Kvakr5	97.8	4.00E-23	28.2	91.5	chromosome	226,996–227,925	309	No	NAD(P)-linked oxidoreductase

Kvakr6	148.3	3.00E-40	31.9	93.0	megaplasmid pP1	180,421–181,410	329	No	NAD(P)-linked oxidoreductase

Table S4. Percent identity matrix of amino acid sequences created through Clustal Omega program.

	**DepA**	**QDDH**	***Kv*ADH3**	***Kv*ADH4**	***Kv*ADH1**	***Kv*ADH2**
DepA	100.00					
QDDH	100.00	100.00				
*Kv*ADH3	54.87	54.87	100.00			
*Kv*ADH4	55.86	55.86	86.49	100.00		
*Kv*ADH1	57.76	57.76	82.31	83.42	100.00	
*Kv*ADH2	55.14	55.14	80.69	81.98	84.14	100.00

Table S5. DNA primers and PCR conditions.

Candidate Gene	PCR Forward Primer	PCR Reverse Primer	Thermocycling Conditions
Kvadh1	GTGGTGGTGGTGGTGTTGCGGCAGGGCGAAGAC	AGGAGATATACCATGCAAGTAACCCCGATCACCGAT	98 °C/2 min; 35 × (98 °C/10 s,72 °C/90 s) ;72 °C/2 min
Kvadh2	GTGGTGGTGGTGGTGCTGCGGCAGTGCAAAGAC	AGGAGATATACCATGCAGGTAACCCCGATTACCGATGA	98 °C/2 min; 5 × (98 °C/10 s,69 °C/30 s,72 °C/60 s) ; 30 × (98 °C/10 s,72 °C/90 s) ;72 °C/2 min
Kvadh3	GTGGTGGTGGTGGTGTTGCTGCGGCAGAGCAAAGAC	AGGAGATATACCATGCAAACCGCCATCACCGATGAA	98 °C/2 min; 35 × (98 °C/10 s,72 °C/90 s) ;72 °C/2 min
Kvadh4	GTGGTGGTGGTGGTGTTGCTGGGGCAGCGCG	AGGAGATATACCATGCAAGTGACCCCCGTCACC	98 °C/2 min; 35 × (98 °C/10 s,72 °C/90 s) ;72 °C/2 min
Kvadh5	AGGAGATATACCATGCAGGATGGTTTCTACACCGCTG	GTGGTGGTGGTGGTGTTCAGCGGTCAGACGATAGACATAGAC	98 °C/2 min; 5 × (98 °C/10 s,69 °C/30 s,72 °C/60 s) ; 30 × (98 °C/10 s,72 °C/90 s) ;72 °C/2 min
Kvadh6	AGGAGATATACCATGAGAGCGACGGCACC	GTGGTGGTGGTGGTGTGGCAGGGCATAGGCGATGAAA	98 °C/2 min; 5 × (98 °C/10 s,69 °C/30 s,72 °C/120 s) ; 30 × (98 °C/10 s,72 °C/120 s) ;72 °C/2 min
Kvadh7	AGGAGATATACCATGCAAATCGCTGATTACACCCCACT	GTGGTGGTGGTGGTGGTTCACATCAGGCAGGGCG	98 °C/2 min; 5 × (98 °C/10 s,70 °C/30 s,72 °C/60 s) ; 30 × (98 °C/10 s,72 °C/90 s) ;72 °C/2 min
Kvadh8	AGGAGATATACCATGCAAATGACGCCGATTACCGACG	GTGGTGGTGGTGGTGGGTCTTTGCGGGGCTCG	98 °C/2 min; 5 × (98 °C/10 s,69 °C/30 s,72 °C/60 s) ; 30 × (98 °C/10 s,72 °C/90 s) ;72 °C/2 min
Kvakr_1	AGGAGATATACCATGATCACCCGTGAACTTGGC	GTGGTGGTGGTGGTGGCGGCCCGAGAATTTCAATG	98 °C/2 min; 5 × (98 °C/10 s,68 °C/30 s,72 °C/40 s) ; 30 × (98 °C/10 s,72 °C/70 s) ;72 °C/2 min
Kvakr_2	AGGAGATATACCATGGAATACCGTAAGCTTGGCAG	GTGGTGGTGGTGGTGGGCTTCGGGCGCG	98 °C/2 min; 5 × (98 °C/10 s,68 °C/30 s,72 °C/40 s) ; 30 × (98 °C/10 s,72 °C/70 s) ;72 °C/2 min
Kvakr_3	AGGAGATATACCATGAAACAGCTGGAACTGGGC	GTGGTGGTGGTGGTGAAAAGGCATCGCATGGGC	98 °C/2 min; 5 × (98 °C/10 s,68 °C/30 s,72 °C/40 s) ; 30 × (98 °C/10 s,72 °C/70 s) ;72 °C/2 min
Kvakr_4	AGGAGATATACCATGACGAACATGATTGCAAAAGTCCC	GTGGTGGTGGTGGTGGACACGCTGCCCCTGC	98 °C/2 min; 5 × (98 °C/10 s,69 °C/30 s,72 °C/40 s) ; 30 × (98 °C/10 s,72 °C/70 s) ;72 °C/2 min
Kvakr_5	AGGAGATATACCATGAAAACCTATACCGTCCCCCA	GTGGTGGTGGTGGTGGGGCAGGGTATGGCCC	98 °C/2 min; 5 × (98 °C/10 s,69 °C/30 s,72 °C/40 s) ; 30 × (98 °C/10 s,72 °C/70 s) ;72 °C/2 min
Kvakr_6	AGGAGATATACCATGAAAACCCGTAAACTTGGTGCG	GTGGTGGTGGTGGTGACGATACGAGTATTGCAGAACGG	98 °C/2 min; 5 × (98 °C/10 s,68 °C/30 s,72 °C/40 s) ; 30 × (98 °C/10 s,72 °C/70 s) ;72 °C/2 min
linearized vector	CATGGTATATCTCCTTCTTAAAGTTAAACAAAATTATTTCTAGAGG	CACCACCACCACCACCACT	98 °C/2 min; 35 × (98 °C/10 s,68 °C/30 s,72 °C/180 s) ;72 °C/4 min

Note: The underlined sequence highlight the 15 bases of homology to the entry vector of pET28a.
